# The Potential of CAR T Cell Therapy in Pancreatic Cancer

**DOI:** 10.3389/fimmu.2018.02166

**Published:** 2018-09-25

**Authors:** Mehmet Akce, Mohammad Y. Zaidi, Edmund K. Waller, Bassel F. El-Rayes, Gregory B. Lesinski

**Affiliations:** ^1^Department of Hematology and Oncology, Winship Cancer Institute, Emory University School of Medicine, Atlanta, GA, United States; ^2^Department of General Surgery, Indiana University, Bloomington, IN, United States; ^3^Division of Surgical Oncology, Department of Surgery, Emory University School of Medicine, Atlanta, GA, United States

**Keywords:** pancreas cancer, mesothelin, adoptive T cell therapy, CAR T cells, genetically engineered T cells

## Abstract

Pancreatic cancer has a dismal prognosis and effective treatment options are limited. It is projected to be the second most common cause of cancer related mortality in the United States by 2030 and there is urgent unmet need for novel systemic treatment options. Immunotherapy with antibodies targeting PD-1, PD-L1, CTLA-4 has not shown clinical activity in unselected pancreatic cancer, emphasizing the need for combination immunotherapy approaches or other therapeutic strategies. As such, chimeric antigen receptor (CAR) T cell therapy represents an emerging therapeutic option for pancreatic cancer. This modality utilizes genetically engineered T cells that are redirected to specific cancer-associated antigens to elicit potent cytotoxic activity. This review summarizes the available preclinical data and highlights early phase clinical trials using CAR T cell approaches in pancreatic cancer, a disease state that is gaining attention as a conduit for cell therapy. Future directions in application of CAR T cell therapy are also considered including its ability to be directed against novel epitopes and combined with other therapeutic regimens.

## Introduction

Pancreatic cancer is the fourth most common cause of cancer related mortality in the United States, and estimates project it to become the second most common cause of cancer related mortality by 2030 (1, 2). Approximately 60% of patients present with advanced disease and median overall survival ranges between 8.5 and 11.1 months with the current therapeutic approaches (3, 4). The majority of patients who present with localized disease eventually develop metastasis. Therefore, development of effective systemic therapies is key in improving the outcomes of patients with pancreatic cancer. The therapeutic options for systemic therapy are limited and clinical activity is at best modest. Immunotherapy with checkpoint inhibitors has emerged as a novel therapeutic option in many solid and hematologic malignancies. However, trials in pancreatic cancer with checkpoint inhibitors have only shown activity in a small subset of patients with micro-satellite instability of their cancer, which increases mutation burden and the likelihood of tumors harboring neoantigens. The limited success of immunotherapy in pancreatic adenocarcinoma is multi-factorial, but certainly influenced by a notoriously immunosuppressive tumor microenvironment (TME) and lack of tumor-associated neo-antigens to stimulate an immune response in most patients. Therefore, systemic therapies for pancreatic cancer remain a significant unmet need.

Adoptive cellular therapy using tumor antigen-specific T cells has endured a remarkable evolution from its roots in the setting of malignant melanoma. The initial development of this approach was led by Dr. Steven Rosenberg and his colleagues in the 1980s using TILs isolated from melanoma patients (5–7). Decades of subsequent translational and clinical research by many talented investigators led to continued refinement of adoptive T cell therapy approaches and the critical factors that can be manipulated to maximize their efficacy. These advances include a multitude of factors including pre-conditioning regimens, optimal *ex vivo* expansion protocols, and co-administration of cytokines, among many others [Reviewed in (7, 8)]. Further refinement of antigen specificity was achieved in the 1990s following the development of gene transfer techniques that enabled introduction of chimeric antigen receptors (CARs) into T cells (7, 9, 10).

This review addresses the potential for CAR T cell therapy in the setting of pancreatic cancer. Herein we summarize both preclinical and early-phase clinical experience in CAR-mediated redirection of T cells. Key antigens of relevance to pancreatic ductal adenocarcinoma (PDAC) are discussed, along with innovative future directions of research occurring in this rapidly moving field.

## CAR-T cells represent a promising therapeutic modality

**Figure 1:**
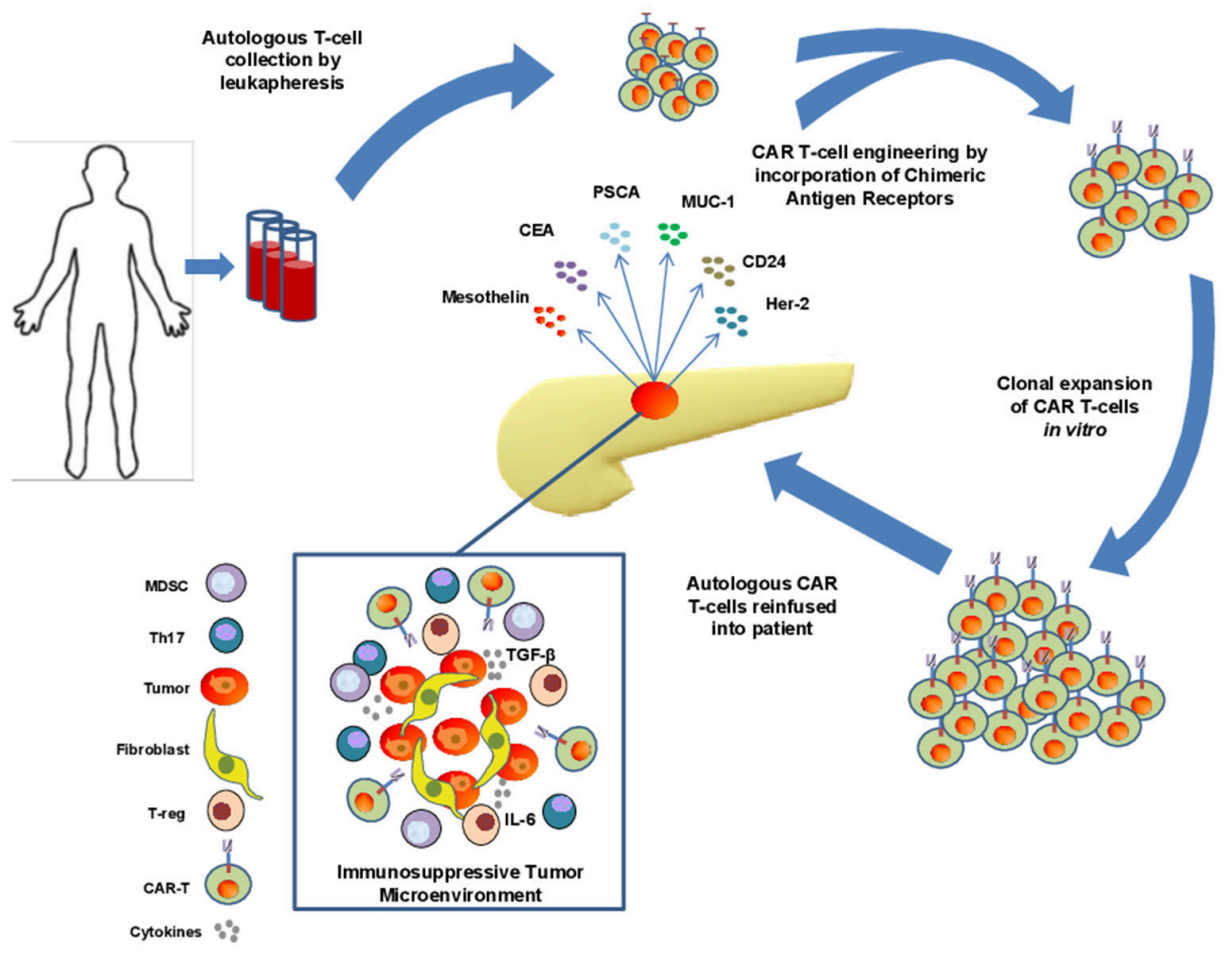
Isolation, engineering, and challenges of CAR T cell therapy in pancreatic adenocarcinoma (PDAC). T cells are collected from peripheral blood of patients with PDAC via leukapheresis and engineered to express chimeric antigen receptors directed toward a specific tumor antigen. These cells are subsequently expanded before reinfusion into patients. Significant challenges exist for these cells to infiltrate the immunosuppressive tumor microenvironement of PDAC including the presence of dense stroma and myofibroblast cells, immunosuppressive cytokines such as IL-6 and TGF-β, and the presence of immunosuppressive immune cell types such as Th17 cells, MDSCs, and suppressive T-regs.

Adoptive transfer of lymphocytes continues to evolve as a treatment modality for advanced cancer. This general approach can leverage the versatility of T cells and their ability to be redirected toward relevant tumor antigens via engineered T cell receptors (TCRs) or CARs. Redirecting cell specificity via CARs represents one sophisticated approach that has gained traction in clinical care of hematologic malignancy. To generate the appropriate cell therapy product, T cells are collected from patient peripheral blood by leukapheresis and redirected to a specific antigen via viral expression of a Chimeric Antigen Receptor (CAR; Figure 1). To date, this approach has been widely utilized as an individualized therapy with genetic modification of autologous T cells from patients, although “off-the-shelf” CAR T cell approaches are beginning to emerge using T cells from allogeneic donors. The CAR constructs, when incorporated into T cells, mimic TCR activation, and redirect specificity and effector function toward an intended antigen, with the important advantage of eliciting recognition in a non-MHC-restricted manner (11).

The design of CARs continues to evolve, whereby the first-generation constructs contained an extracellular ligand-binding domain, such as a single chain variable fragment (scFv) that is directed toward a specific antigen, along with the CD3ζ or Fc receptor γ signaling domain (10, 11). Subsequent second generation or third generation CARs contain one or more costimulatory domains, respectively such as CD28, 4-1BB, ICOS or OX40 to selectively modify the function and/or persistence of the resultant CAR T cells (12–17). For example, inclusion of the CD28 domain can afford a more rapid expansion of the CAR T cells, while the 41BB domain is typically used to enhance *in vivo* persistence of the cells, albeit at a slower rate of expansion. These particular domains may also lead to a differential effect on redirected CD4^+^ or CD8^+^ T cell subsets. In a general sense, the CD28 molecule is recognized to more selectively promote expansion of naïve and CD4^+^ T cells, while the 41BB domain is more relevant to facilitating expansion of memory and CD8^+^ T cell subsets (17). Finally, development of antigen-redirected T cells has already entered the realm of an imaginative transition to utilizing fourth generation CAR constructs. These include highly sophisticated engineering that incorporates the ability of redirected T cells to produce immunomodulatory cytokines (e.g., IL-12, IL-18, IL-21) or antibodies (e.g., anti-PD-1), optimize trafficking via expression of chemokine receptors, and converting immune suppressive signals rich within the tumor microenvironment into activating signals that further enhance T cell activity. These and other constructs incorporating safety-intended suicide switches and receptors conferring bi-specificity and other signaling domains are in the early phases of both pre-clinical and clinical testing. These fourth generation CAR constructs and their application in solid tumors is the topic of a detailed, informative review by Knochelmann et al. (15).

Once CAR T cells are generated and undergo clonal expansion in culture, cells can be infused into patients to achieve antigen-directed, T cell mediated antitumor immune responses. In order to maximize *in vivo* expansion and decrease inhibitory lymphocytes that may persist systemically, patients are given a chemotherapy pre-conditioning regimen for lymphodepletion prior to infusion of CAR T cells (13, 14). Bulk T cell populations are typically reinfused as a cellular product for yield and logistical reasons, however the relative ratio of CD4^+^ vs. CD8^+^ T cells that are redirected toward antigen via CAR constructs may impact both the effector function and persistence of the cells. This emphasizes the complexity of implementing CAR T cell therapeutic approaches, and from a biological perspective, the importance of T cell help in generating effective antitumor immune responses.

Although dramatic efficacy can be achieved in patients following CAR T cell therapy, there is a risk for serious adverse events. In particular, cytokine release syndrome and neurological toxicity have been observed in patients, and illustrate the consequences of potent immune recognition of antigenic targets and the resulting robust immune response. In the context of pediatric ALL and other disease settings, administration of antibodies that block the interleukin-6 (IL-6) receptor are effective for reversal of the cytokine release syndrome (18), and allow patients to tolerate CAR T cell therapy. A provocative report by Fraietta et al. further showed that serum IL-6 correlated with CAR T cell expansion and that IL-6/STAT3 signaling may be important for proliferation of the redirected T cells in the setting of chronic lymphocytic leukemia (19). These data highlight the complexity of cytokine signaling in mediating toxicity and potential response related to CAR T cell therapy. More recent pre-clinical studies by Giavridis et al. report that IL-1 blockade may also be an effective means to control this cytokine release syndrome secondary to CAR T cell therapy (20). However, the concerns with neurotoxicity highlight the need for careful antigen selection, and patient monitoring during the course of these therapies.

## Clinical efficacy of car T cell therapy in hematologic malignancy has provided a firm foundation for growth

A wealth of clinical experience with CAR T cell therapy has been derived from hematologic malignancy. Clinical efficacy of CAR T cell therapy was shown when Kochenderfer et al. reported a partial response with anti-CD19 CAR T cell therapy in a patient with treatment-refractory stage IVB follicular lymphoma (21). This was the first reported patient who achieved a clinical response with anti-CD19 CAR T cell therapy and it lasted 8 months before the patient developed progressive disease. Porter et al. utilized anti-CD19 CAR T cell therapy in a patient with refractory CLL and achieved a complete remission 3 weeks after treatment, which persisted for at least 10 months (22). CAR T cells persisted for 6 months at high levels in the peripheral blood and bone marrow. Two other patients treated with the same regimen achieved clinical response. These early findings prompted further development of a prospective studies in hematologic malignancies and produced overall response rates ranging from 52 to 92%. Complete response rates ranging from 43 to 90% were reported in subsequent clinical trials with anti-CD19 CAR T cell therapy in treatment refractory advanced lymphomas (18, 23–29). The ZUMA-1 and 2 trials, and ongoing ZUMA-3 and 4 trials reported high and durable response rates in patients with relapsed refractory large B cell lymphomas and with relapsed refractory pre-B cell ALL. Additionally, the phase II ELIANA trial of CTL019 CAR T cell therapy reported 75% relapse free probability at 6 months after remission, 89% probability of survival at 6 months, and 79% probability of survival at 12 months in pediatric and young adult patients with relapsed and refractory ALL (26, 30–33). This clinical trial provided the foundation for development of Tisagenlecleucel, CTL019, (Kymriah) which as a result was approved by the FDA for treatment of relapsed refractory B-cell precursor ALL in pediatric and young adult patients. This was followed by FDA-approval of axicabtagene ciloleucel (axi-cel; Yescarta), which targets CD19 in adult patients with relapsed refractory large B cell lymphoma after 2 lines of prior therapy (34).

## Paving the road for car T cells in PDAC with preclinical studies

**Figure 2:**
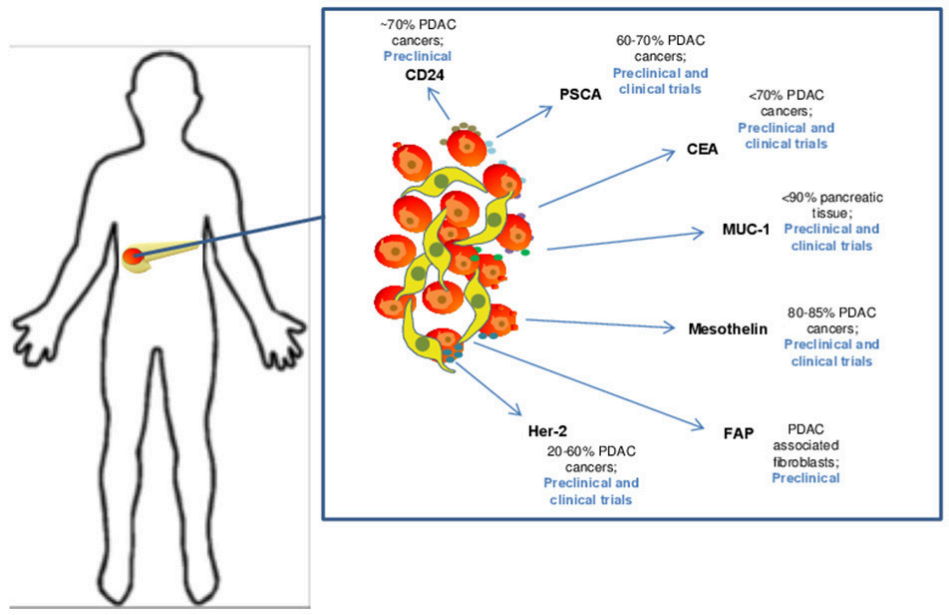
Candidate target antigens for CAR T cell therapy in pancreatic ductal adenocarcinoma. Several target antigens for CAR T cell therapy have been identified and are being studied in both preclinical and clinical trails. These antigens are expressed at varying levels on PDAC cells and include CD24 (35), Prostate stem cell antigen (PSCA) (36), CEA (37), MUC-1 (38), Mesothelin (39), FAP (40), and Her-2 (41).

A number of published studies demonstrate efficacy of CAR T cell therapy platforms in preclinical models of pancreatic cancer. The results to date are derived from multiple *in vivo* models, including transplantable human tumors, patient-derived xenografts, and in a more limited number of cases, immune competent mice with spontaneously-arising, or transplanted syngeneic tumors. One priority for the field remains the need to discover and validate new, bona-fide pancreatic cancer-specific antigens to minimize potential on-target, off-tumor adverse events. However, the existing data with several candidate antigens has infused some momentum into the field (Figure 2). For example, pre-clinical work on CAR T cells in pancreatic cancer targeting the tumor antigen mesothelin subsequently led to human studies. Mesothelin is a glycoprotein over-expressed on a variety of tumor cells including pancreatic cancer. This antigen is present on the cell surface, first as a 69-kDa protein, that later becomes cleaved via the furin protease into a soluble 32 kDa N-terminal fragment and a 40-kDa C-terminal fragment that remains membrane bound (44, 45). From a functional standpoint, data indicate mesothelin positively regulates tumor growth and invasion. The protein has relatively limited expression in normal tissue, where it is localized to mesothelial cells, including cells of the pleura, pericardium, peritoneum, fallopian tubes, trachea, tonsils, and cornea where its function is unclear (44, 46–48). One intriguing observation qualifying its role as an antigen of interest is that mesothelin-specific CD4^+^ and CD8^+^ T cell reactivity is evident in patients with pancreatic cancer (48–50). Mesothelin-directed CAR T cell therapy has been used widely across pre-clinical models in a variety of malignancies (51–54), including elegant studies in pancreatic cancer by Beatty et al. whereby T cells were redirected via mRNA electroporation of mesothelin-targeted CAR with CD3z and 4-1BB signaling domains to elicit a transient CAR expression for safety (55).

An assortment of preclinical studies targeting antigens in preclinical models of PDAC continue to emerge (Figure 2). Among these are interesting data in which CAR T cells were redirected against CEA (CEACAM5), a long-standing antigen of interest for immune response in advanced gastrointestinal malignancy. The expression profile of CEA has made it a target for vaccine trials (56, 57). It is expressed in up to 70% of pancreatic cancers, leading to its potential as a tumor specific antigenic target. In a murine model of PDAC, CEA-redirected CAR T cells produced long-term anti-tumor responses with no evidence of damage to normal tissues with lower levels of CEA expression (58). Targeting of other tumor antigens including PSCA, HER2 neu CD24 and MUC1 has also demonstrated impressive activity in preclinical PDAC models (36, 59–62). Other innovative approaches for redirecting T cells are in development as our understanding into the complexity of tumor antigens in PDAC improves. For example, a recent study by Posey et al. directly addressed the poor expression of cancer-specific antigens on the cell surface by developing a CAR that recognized a Th glycoform of the MUC1 antigen. This creative work showed CAR T cells redirected against this aberrantly glycosylated antigen were efficacious in xenograft models of PDAC and leukemia (62). These data highlight the potential for re-invigorated antigen discovery when considering finer structure of proteins and their modifications that can be preferentially expressed on PDAC cells.

## Overcoming suppressive “roadblocks” for effective car T cell therapy in pancreatic cancer

One notable challenge for CAR T cell therapy in preclinical models of PDAC is the inherent histologic properties and profound immunosuppressive capacity of this tumor type (Figure 1). For example, the desmoplastic reaction present in a majority of pancreatic tumors leads to a dense stroma that in theory, can serve as a physical barrier for drug delivery and penetration of endogenous or engineered T effector cells. Various components of the stroma can also serve as a source of abundant inhibitory cytokines, including IL-6 (63). In the context of other cytokines in the tumor microenvironment, such as TGF-β or IL-10 (42, 64) a cytokine milieu emerges which promotes expansion and enhanced suppressive function of cells such as T-regs, Th17 and myeloid derived suppressor cells (MDSC). Together these soluble and cellular factors can limit the function of any cytotoxic T cells that may be directed toward tumor antigens.

Overcoming this immunosuppressive microenvironment is a potential strategy to improve CAR T cell survival, trafficking and persistence in PDAC, and numerous pre-clinical studies addressing this concept are underway. For example, heparanase is under investigation as a means to overcome the physical barriers of the desmoplastic reaction, and in preclinical studies, was shown to increase antitumor activity and tumor infiltration of CAR redirected T lymphocytes (65). To address inefficient trafficking of CAR T cells into the tumor microenvironment, several studies have employed targeted approaches redirecting these cells to prominent cell surface molecules that are of relevance for pancreatic cancer. For example, the chemokine receptor CCR2 was successfully transduced into mesoCAR T cells, which increased T cell infiltration and antitumor activity compared to conventional mesoCAR T cell administration in immunodeficient mice with large mesothelin expressing tumors (66). Likewise, fibroblast activation protein (FAP) has been leveraged as a potential target antigen in the context of CAR T cells. This antigen is particularly unique given its expression on myofibroblast cells present within the pancreatic stroma. In an eloquent proof of principle study, Tran et al. developed a novel CAR T cell approach whereby cells were redirected to interact with FAP, and adoptively transferred in to mouse models bearing a variety of subcutaneous tumors including pancreatic adenocarcinoma. This approach was associated with cachexia and lethal bone toxicity, which most likely would limit its utilization as a universal target with CAR T cell therapy (40).

Another principal mediator of immune suppression is upregulation of immune checkpoint receptors including PD-1 on the surface of antigen-experienced T cells. Increased expression of PD-1 is associated with T cell exhaustion, and blocking the interaction of PD-1 with its ligands PD-L1 and PD-L2 has shown impressive clinical activity in oncology, leading to FDA-approval for several antibodies targeting this immunosuppressive axis (67). Similarly, CAR T cells upon infusion can upregulate the inhibitory, PD-1 receptor on their surface (68). Similar to unmanipulated T lymphocytes, PD-1 upregulation on CAR T cells can signify exhaustion of effector function, limiting the efficacy of CAR T cell based therapy. These observations provide rationale to combine CAR T cell therapy with antibodies targeting the PD-1/PD-L1 axis (69). This concept is being explored clinically in combination immunotherapy trials, and in sophisticated, fourth-generation CAR design strategies that allow for genetic ablation of PD-1 in antigen redirected cells (70–72) or even production of PD-1/PD-L1-targeted agents by the modified T cells themselves (73, 74). These advanced design approaches highlight the adaptability of T cells as a therapeutic platform and how they can be manipulated to overcome redundant mechanisms of immune suppression in cancer.

## Clincial experience with car T cells in pancreatic cancer

Preliminary results of case reports using CAR T cell therapy in pancreas and other non-hematologic malignancies encouraged development of multiple clinical trials in pancreatic cancer with CAR T cell therapy. Multiple different antigens specific to the tumor are being targeted in current ongoing clinical trials in non-hematologic malignancies including pancreas cancer (Table 1). Several antigens including CEA, HER2, MUC1, CD133, prostate stem cell antigen (PSCA) and mesothelin are prominent targets using this approach in pancreatic cancer (Figure 1). The challenges in developing CAR T cells in non-hematologic malignancies include lack of cell surface tumor-specific molecules that could be targeted by genetically engineered CAR T cells (75). The expression of the targeted antigen in normal and tumor tissues raises concern regarding potential off target toxicity (76, 77). Another challenge present clinically is the profound degree of immunosuppression that limits both the persistence of CAR T cells and their ability to effectively traffic to the tumor sites. Lymphodepletion has been shown in prior trials to enhance expansion, cytokine release, and persistence of the CAR T cells. Cyclophosphamide alone or in combination with fludarabine has been utilized in various CAR T cell therapy trials in hematologic malignancies as preconditioning regimens in order to achieve lymphodepletion prior to CAR T cell therapy. The success of CAR T cells depends on their ability to expand *ex vivo* while loss of central memory and naïve T cell subsets can be seen in heavily treated lymphoma patients due to multiple rounds of cytotoxic chemotherapy. This may also be a factor of relevance in patients with PDAC who may be candidates for experimental trials with CAR T cells, as most patients will have received systemic chemotherapy as standard of care. A recent report has shown that the quality of patient T cells could be a variable in the success of CAR T cell manufacturing, particularly loss of CD27/CD28 expression in heavily pre-treated lymphoma patients (78). Considering many lines of prior systemic therapy in non-hematologic malignancies prior to CAR T cell therapies similar challenges could be encountered in this area as well.

**Table 1:** Recently completed and ongoing clinical trials with CAR T cell therapy in pancreatic cancer.

**Diagnosis**	**Target antigen/biologic**	**Preconditioning**	**Route**	**Study phase**	**Institution**	**Study ID**	**Status**	**Est. stop**
CEA^+^ PDAC w/liver mets and persistent disease post ≥1 line of chemotherapy	CEA/anti-CEA CAR T-cell	–	Hepatic artery infusion	I	Roger Williams Cancer Center, USA	NCT02850536	Active, not recruiting	2018
CEA^+^ cancer (including PDAC), refractory or relapsed	CEA/anti-CEA CAR T-cell	–	IV	I	Southwest Hospital, China	NCT02349724	Recruiting	2019
CD133^+^ liver, pancreas, breast, ovarian, CRC, brain cancers and acute leukemia	CD133/anti-CD133 CAR T cells	–	–	I	Chinese PLA General Hospital, China	NCT02541370	Recruiting	2018
CD70^+^ cancer (including PDAC)	CD70/anti-CD70 CAR T cells	Cy/Flu	IV	I/II	National Cancer Institute, USA	NCT02830724	Recruiting	2021
Claudin 18.2^+^ advanced gastric or PDAC	CLD18/anti-CLD18 CAR T cells	–	IV	Pilot	Changhai Hospital, China	NCT03159819	Recruiting	2021
EpCAM^+^ cancer (including PDAC), refractory or relapsed	EpCAM/anti-EpCAM CAR T cells	–	IV	I/II	First Affiliated Hospital of Chengdu Medical College, China	NCT03013712	Recruiting	2020
HER-2^+^ cancer (including PDAC), refractory or relapsed	HER-2/anti-HER2 CAR T cells	–	IV	I/II	Southwest Hospital, China	NCT02713984	Recruiting	2019
Unresectable/mestatic cancer post 1st-line chemotherapy	Mesothelin and CD19/CART-meso-19 T cells	CP	IV	I	UPenn, USA	NCT02465983	Completed	2017
Mesothelin^+^ cancer, unresectable and persistent post 1st-line chemotherapy	Mesothelin/TAI-meso-CAR T cells	CP	Transarterial infusion	I	Renji Hospital, China	NCT02706782	Recruiting	2018
PDAC, metastatic and refractory	Mesothelin/anto-mesothelin immunoreceptor SS1	–	IV	I	UPenn, USA	NCT01897415	Completed	2017
PDAC	Mesothelin/HuCART-meso cells	–	–	I	UPenn, USA	NCT03323944	Active, not recruiting	2017
Mesothelin^+^ cancer (including PDAC)	Mesothelin/anti-mesothelin CAR T cells	Cy/Flu	IV	I/II	National Cancer Institute, USA	NCT01583686	Recruiting	2029
PDAC, metastatic and relapsed	Mesothelin,PSCA,CEA, HER2,MUC1, EGFRvIII and others/Mesothelin/PSCA/CEA/ HER2/MUC1/,EGFRvIII and other CAR T cells	–	IV	I	First Affiliated Hospital of Harbin Medical University, China	NCT03267173	Recruiting	2019
PDAC, HCC, CRC, metastatic	Mesothelin for PDAC, GPC3 for HCC, CEA for CRC/targeted CAR T cells	–	Vascular interventional therapy or by intra-tumor injection	I/II	Shanghai GeneChem Co., Ltd. China	NCT02959151	Recruiting	2018
PDAC, ovarian carcinoma and pleural mesothelioma	Mesothelin/anti-mesothelin CAR T cells	CP	IV	I	China Meitan General Hospital, China	NCT02930993	Recruiting	2019
PDAC, malignant mesothelioma, ovarian cancer, endometrial cancer, triple negative breast cancer, other mesothelin^+^ tumors	Mesothelin/anti-meso-CAR vector transduced T cells	–	–	I	Chinese PLA General Hospital, China	NCT02580747	Recruiting	2018
PDAC, ovarian cancer, pleural mesothelioma	Mesothelin/Anti-mesothelin CAR T cells	–	–	I	UPenn, USA	NCT02159716	Completed	2015
PDAC, no curative options after resection, (R1 resection excluded) Enrolls certain other solid tumors	MUC-1/Anti-MUC1 CAR T cells	–	–	I/II	Hefei Binhu Hospital, China	NCT02587689	Recruiting	2018
PDAC, unresectable, treatment naïve or refractory	PSCA/PSCA-CAR T- cells	–	IV	I	Baylor Sammons Cancer Center, USA	NCT02744287	Recruiting	2020

*CEA, carcinoembryonic antigen; PDAC, pancreatic ductal adenocarcinoma; IV, intravenous; CRC, colorectal cancer; Cy, cyclosporine; Flu, fludarabine; CP, cyclophosphamide; HCC, hepatocellular carcinoma; UPenn, University of Pennsylvania*.

Data from pre-clinical studies have permitted translation of CAR T cell therapy into several clinical trials for patients with pancreatic cancer (Table 1). Largely, target antigens used in patient trials have overlapped with the pre-clinical studies leading up to them. To date, the most experience with CAR T cells in PDAC patients has been gained through targeting mesothelin. Approximately 80–85% of pancreatic carcinomas express mesothelin and as a result, it has become a prominent target for CAR T cell therapy trials (39, 44). At the time of this review article, 10 separate clinical trials using CAR T cells directed against mesothelin have been completed or are in progress. The first results using mesothelin-directed CAR T cells (CARTmeso cells) were reported by Beatty et al. in a case report from two patients, one with malignant pleural mesothelioma (MPM) and a second with pancreatic cancer (55). In this trial, a major goal was to ensure safety given potential for expression of target antigens on normal tissues. To accomplish this, the authors developed a strategy for transient CAR expression via mRNA electroporation encoding an anti-mesothelin ss1 scFv CAR. Interestingly, in the patient with pancreatic cancer, the T cells were obtained from his monozygotic twin brother, activated, expanded with mRNA electroporation encoding the CAR. The CARTmeso cells were administered as eight doses of intravenous infusion followed by two intratumoral injections for the pancreatic cancer patient. No lymphodepleting therapy was given to either patient. Results indicated the CARTmeso transgene was detectable in peripheral blood in both patients after intravenous CARTmeso administration at pre-specified time points per the study; in the ascitic fluid 3 days after the initial intravenous infusion and 13 days after first intratumoral injection in the patient with pancreatic cancer. CARTmeso transcripts were also detected in the pre and post-pancreatic tumor biopsy tissue in the patient with the first intratumoral injection of CARTmeso cells. This finding indicated CARTmeso cell trafficking into the tumor tissue indeed occurred following intravenous administration. The patient with MPM achieved partial response, which lasted 6 months, and the patient with pancreatic cancer achieved stable disease.

In a more recent phase I study of CARTmeso cells by the same group, 6 patients with treatment refractory metastatic pancreatic cancer were administered CARTmeso cells intravenously 3 times per week for 3 weeks. In this trial, no dose limiting toxicity, cytokine release syndrome or neurological complications were encountered, while stable disease was reported in two of the patients (79). These findings were complemented by decreased FDG uptake on PET computed tomography, suggestive of activity against the tumor in these patients. This initial encouraging safety data with a more conservative, transient expression approach of CAR targeting mesothelin has prompted further attempts at targeting this antigen using more stable introduction of lentiviral constructs into T cells (NCT02159716). Several other trials are currently open to patients with mesothelin-expressing PDAC to further evaluate CARs directed at this antigen (NCT01583686, NCT02465983) (48).

Clinical experience with targeting other antigens via CAR T cells continues to emerge (Table 1). For example, multiple clinical trials are underway with CEA-directed CAR T cell therapy that include patients with pancreatic cancer (NCT02850536, NCT02349724). However, existing data with targeting CEA emphasize cautionary discretion is needed in subsequent clinical studies. In a feasibility study, anti-CEA CAR T cell therapy resulted in significant respiratory toxicities in patients with advanced CEACAM5^+^ gastrointestinal malignancies, including pancreatic cancer. These results necessitated premature closure of the study (80). Clinical trials with CAR T cells redirected against HER2 neu have also gained traction in the setting of PDAC, yet will proceed with caution given potential for toxicity. Namely, one clinical study was terminated early, after the first patient (with colorectal cancer) died as a result of pulmonary toxicity, cytokine storm, and multiorgan failure arising following infusion of the HER2 neu targeted CAR T cells (81). (NCT00924287) Other trials are ongoing in HER2 neu positive malignancies with CAR T cells redirected against this antigen (NCT02713984, NCT00889954) that will be informative once completed for assessment of both safety and potential for toxicity.

Equipping CAR T cells to engage tumors via specificity for other antigens in the setting of PDAC will continue to progress into clinical trials. This is a rapidly moving field that has extended beyond mesothelin, CEA and HER2 neu and into additional antigens including PSCA (36, 59, 60), and in MUC-1 (Table 1), among others. Of paramount importance will be careful consideration of on-target toxicities and antitumor efficacy, given the limited experience of adapting CAR T cells into solid tumor oncology.

While our understanding of CAR T cell-mediated adverse events remains in its early stages, combination immunotherapy approaches continue to enter clinical trials at a remarkably rapid pace (82). This momentum has been influenced by the success of immune checkpoint blockade, particularly with antibodies targeting PD-1 and CTLA-4 (67). There is a high level of interest in the field to develop treatment strategies that administer these antibodies, which can reverse T cell exhaustion with the antigen-specificity of CAR T cell therapy. Although a majority of this work is occurring in hematologic malignancy, its application to PDAC or other solid tumors is becoming accelerated. Novel studies are ongoing utilizing autologous CAR T cells redirected to recognize mesothelin and simultaneously engineered to express antibodies targeting PD-1 and CTLA-4 for recurrent and refractory non-hematologic malignancies (NCT03182803, NCT03030001). Another clinical trial is recruiting patients with non-hematologic tumors to evaluate the novel combination of autologous MUC1-redirected CAR T cells engineered to express antibodies targeting CTLA-4 and PD-1 (NCT03179007). Finally, sophisticated gene editing technologies such as CRISPR/Cas9 are being utilized to further engineer CAR T cells that lack genes encoding factors such as PD-1 that mediate an exhausted phenotype and function (71, 72) or alternatively aid in the design of universal, “off-the-shelf” CAR T cell approaches that lack self-recognition factors (83, 84). Other gene engineering strategies are also in development which incorporate suicide genes to improve the safety of these cell therapy strategies so that the therapeutic effect can be turned on or off, on demand (85).

## Time for custom design: future advances for car T cell therapy in pancreatic cancer

**Figure 3:**
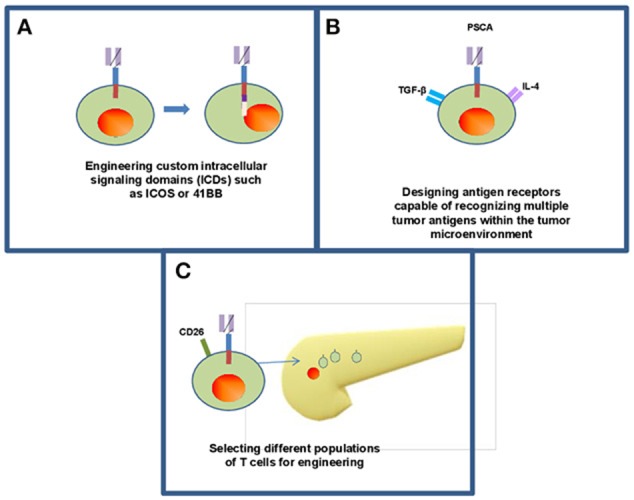
Frontiers in CAR T cell therapy for pancreatic ductal adenocarcinoma. Continuing research within CAR T cell therapy for solid organ malignancy has opened new methods for engineering T cells including enhancing intracellular signaling domains **(A)** to enhance persistence of CAR T cells within the body as well as efficacy against tumors. **(B)** Some groups have designed CARs that recognize multiple antigens within the tumor microenvironment while maintaining their antitumor activity within the body (42). **(C)** There is opportunity to utilize individual phenotypically defined populations of T cells including CD26 high cells (43).

Further advances in customizing the design of CAR T cells continues to emerge and may overcome challenges related to persistence, features of the tumor microenvironment and optimizing phenotype (Figure 3). Guedan et al. addressed some of these challenges by studying how specific intracellular signaling domains (ICDs) such as ICOS or 41BB can be leveraged to enhance the persistence and effector phenotype, respectively (14). These data suggest CAR T cells can be customized to overcome limitations simply by adjusting the ICD of choice. Likewise, our understanding of the optimal cellular compartment to use as a “starting point” for generating CAR T cells continues to mature. While many approaches involve redirecting CD8^+^ T cells to take advantage of their effector function by redirecting mixed CD4^+^ and CD8^+^ populations or even more precise phenotypes such as T cells expressing high levels of the CD26 surface marker (43). These CD26^high^ cells are apparently licensed with a natural capacity to traffic to and elicit antitumor activity in solid tumors including PDAC as shown in preclinical models. Other phenotypically distinct T cell subsets may also be well-suited for use in the setting of CAR T cell therapy. Other subsets including tissue-resident, stem-like T cells could also be considered as candidate cellular templates for CAR-mediated modification. In particular, a CD8^+^ T cell subpopulation expressing CXCR5 and the transcription factor, TCF-1 has been identified in mice that gives rise to new CD8^+^ effector cells from models of chronic LCMV infection and in tumors (86). In theory, these cells could be adaptable as a platform upon which to facilitate redirection of antigen specificity and *in vivo* expansion. Finally, CAR T cells are being designed that capitalize upon these abundant immunosuppressive features within the PDAC microenvironment that in theory can be leveraged as a switch to engage the full cytotoxic potential of CAR T cells. In a clever report, Sukumaran et al. rendered T cells responsive to immunologic features present exclusively at the tumor site by using a trio of CARs that recognize PSCA, TGF-β, and IL-4. These cells were capable of transmitting signals needed for activation, co-stimulation and cytokine support. T cells redirected via these novel constructs elicited antitumor activity against PDAC *in vivo* that was accompanied by T cell expansion and persistence (42). Other preclinical studies continue to provide a foundation for combinatorial therapy involving CAR T cells in PDAC, including combinatorial approaches with innate immune agonists such as STING (87), oncolytic viruses (88), or using FITC-labeled, tumor-directed antibodies to increase CAR T cell recognition (89).

## Conclusions

The impressive results of CAR T cell therapy in hematologic malignancies and preliminary positive outcomes in non-hematologic tumors including pancreatic cancer prompted multiple clinical trials with CAR T cell therapy in this disease setting. Further understanding of the tumor microenvironment, improving off target effects of CAR T cell therapy, reasons of failures with single agent immunotherapy agents, and incorporation of novel agents in combination with CAR T cell therapy may help accomplish effective treatment outcomes in pancreatic cancer. While the extension of CAR T cell therapy into PDAC is just beginning, the field is moving rapidly, and embracing innovative technologies to mitigate potential toxicity and elicit antigen-direct tumor killing.

## Author contributions

MA, GL, and BE-R study concepts. MA, GL, and BE-R study design. MA, GL, BE-R, and EW quality control of data and algorithms. MA, GL, MZ, and BE-R manuscript preparation. MA, GL, BE-R, MZ, and EW manuscript editing. MA, MZ, GL, BE-R, and EW manuscript review.

### Conflict of interest statement

GL, Consultant, ProDa Biotech, LLC, and research support from Merck, Inc.; Vaccinex, Inc. BE-R, Research support from Novartis, BMS, and Merck. The remaining authors declare that the research was conducted in the absence of any commercial or financial relationships that could be construed as a potential conflict of interest.
